# Identification of bacteria in enrichment cultures of sulfate reducers in the Cariaco Basin water column employing Denaturing Gradient Gel Electrophoresis of 16S ribosomal RNA gene fragments

**DOI:** 10.1186/2046-9063-9-17

**Published:** 2013-08-28

**Authors:** Lorelei Bozo-Hurtado, M Alexandra García-Amado, Andrei Chistoserdov, Ramon Varela, J Jesus Narvaez, Rita Colwell, Paula Suárez

**Affiliations:** 1 Departamento de Biología de Organismos, Universidad Simón Bolívar, Caracas, Venezuela; 2 Centro de Biofísica y Bioquímica, Instituto Venezolano de Investigaciones Científicas, Caracas, Venezuela; 3 Department of Biology, University of Louisiana at Lafayette, Lafayette, Louisiana, USA; 4 EDIMAR, Fundación La Salle, Margarita, Venezuela; 5 University of Maryland, College Park, Maryland, USA

**Keywords:** SRB, Cariaco Basin, *Desulfovibrio*, *Vibrio*, DGGE, Culture

## Abstract

**Background:**

The Cariaco Basin is characterized by pronounced and predictable vertical layering of microbial communities dominated by reduced sulfur species at and below the redox transition zone. Marine water samples were collected in May, 2005 and 2006, at the sampling stations A (10°30′ N, 64°40′ W), B (10°40′ N, 64°45′ W) and D (10°43’N, 64°32’W) from different depths, including surface, redox interface, and anoxic zones. In order to enrich for sulfate reducing bacteria (SRB), water samples were inoculated into anaerobic media amended with lactate or acetate as carbon source. To analyze the composition of enrichment cultures, we performed DNA extraction, PCR-DGGE, and sequencing of selected bands.

**Results:**

DGGE results indicate that many bacterial genera were present that are associated with the sulfur cycle, including *Desulfovibrio* spp., as well a**s** heterotrophs belonging to *Vibrio*, *Enterobacter*, *Shewanella*, *Fusobacterium*, *Marinifilum*, *Mariniliabilia*, and *Spirochaeta*. These bacterial populations are related to sulfur coupling and carbon cycles in an environment of variable redox conditions and oxygen availability.

**Conclusions:**

In our studies, we found an association of SRB-like *Desulfovibrio* with *Vibrio* species and other genera that have a previously defined relevant role in sulfur transformation and coupling of carbon and sulfur cycles in an environment where there are variable redox conditions and oxygen availability. This study provides new information about microbial species that were culturable on media for SRB at anaerobic conditions at several locations and water depths in the Cariaco Basin.

## Background

The Cariaco system is a depression located on the northern continental shelf of Venezuela in the Caribbean Sea and is largest true marine permanently anoxic marine water body in the world. The Basin, 160 km long and 50 km wide, is divided into two sub-basins, each with a maximum depth of 1400 m and separated by a saddle at 900 m water depth. Based on redox conditions and oxygen content, the basin is divided into three layers: oxic (~ 0-250 m); redox transition (~ 250–450 m); and anoxic (~ 450 to 1400 m) [1-4]. The basin water column is characterized by a pronounced vertical layering of microbial communities. The oxic layer possesses the most complex trophic structure, dominated by processes such as photosynthesis, aerobic heterotrophy and nitrification. The redox transition zone is biogeochemically stratified, appears less complex and predominant processes are chemoautotrophy, fermentation, denitrification, and anaerobic respiration. The anoxic zone presumably has the simplest trophic structure that appears to be supported by fermentation, sulfate reduction, methanogenesis and anaerobic methane oxidation [3]. Many studies have been conducted in the Cariaco Basin to understand how microorganisms are distributed in the stratified water column environment and how they influence geochemical processes [1,3,5]. Interestingly, high levels of sulfide present in the Cariaco Basin have been attributed to biological sulfate reduction [6]. This is the first attempt to identify bacteria related with sulfate reduction using enrichments cultures for the Cariaco Basin.

Several studies have been published describing the microbial community associated with the Cariaco Basin sulfur cycle. Tuttle and Jannasch (1973) isolated several sulfide and thiosulfate-oxidizing bacteria and Morris *et al*. (1985) isolated *Alteromonas* sp*.* from thiosulfate-containing enrichment cultures [7,8]. With the development of molecular biology, culture-independent methods have been used to detect SRB populations in the Cariaco Basin [1,5,9]. To explore the diversity of bacteria in the Cariaco Basin involved in sulfate reduction, we used SRB enrichment culture complemented with identification of the enriched bacteria by gradient gel electrophoresis (DGGE).

## Results

Depth profiles of temperature, salinity, and dissolved oxygen in the water column at all stations for two years are shown in Table 1. Temperatures varied from 17.10 to 23.85°C, with the greater variation between the surface and interface zone. Salinity was stable throughout the water column at all depths and stations sampled, ranging between 36.26 to 36.88 PSU. Dissolved O_2_ concentration peaked at 40 m (4.009 mL/L) and declined dramatically at depths below 200 m. The maximum measured sulfide concentration was obtained at the greatest depth for all stations during both years of sampling. These physicochemical patterns are typical of the Cariaco Basin, showing redox zonation and similar to those reported by other authors [2,4,6-12].

**Table 1:** Physico-chemical parameters measured in the Cariaco Basin during the study

**Year 2005**						
**Station**	**Zone**	**Depth (m)**	**Temperature (°C)**	**Salinity (PSU)**	**Dissolved O**_**2**_**(mL/L)**	*H_**2**_**S (μM)**
A	Oxic	230	17.90	36.43	0.0738^†^	ND
	Interface	270	17.69	36.39	0.0688	1.22
	Anoxic	900	17.03	36.26	0.0653	51.47
B	Oxic	235	17.83	36.42	0.0711^†^	ND
	Interface	275	17.63	36.38	0.0674	0.69
	Anoxic	670	17.06	36.27	0.0646	ND
**Year 2006**						
Station	Zone	Depth (m)	Temperature (°C)	Salinity (PSU)	Dissolved O_2_ (mL/L)	*H_2_S (μM)
A	Oxic	100	21.08	36.79	3.2116	ND
	Interface	300	17.64	36.36	0.0157	5.39
	Anoxic	400	17.46	36.33	0.0113	14.3
		500	17.23	36.29	0.0113	25.55
B	Oxic	215	17.97	36.41	0.0245^†^	ND
	Interface	260	17.75	36.38	0.0122	6.62
		290	17.70	36.37	0.0129	7.07
	Anoxic	325	17.57	36.35	0.0131	11.09
		640	17.10	36.26	0.0186	38.46
D	Oxic	40	23.85	36.88	4.0087	ND
		180	18.18	36.45	0.5738	ND
	Interface	270	17.80	36.39	0.0135	0.21
	Anoxic	365	17.52	36.34	0.0197	13.31
		500	17.40	36.33	0.0196	18.86

*Data from http://www.imars.usf.edu/CAR/.

^**†**^ Low DO values in oxic zones over the limit of interface layer where DO decays and H_2_S increases.

*PSU*: Practical Salinity Units.

*ND*: Not determined.

### Bacteria enrichments in SRB media

**Figure 1:**
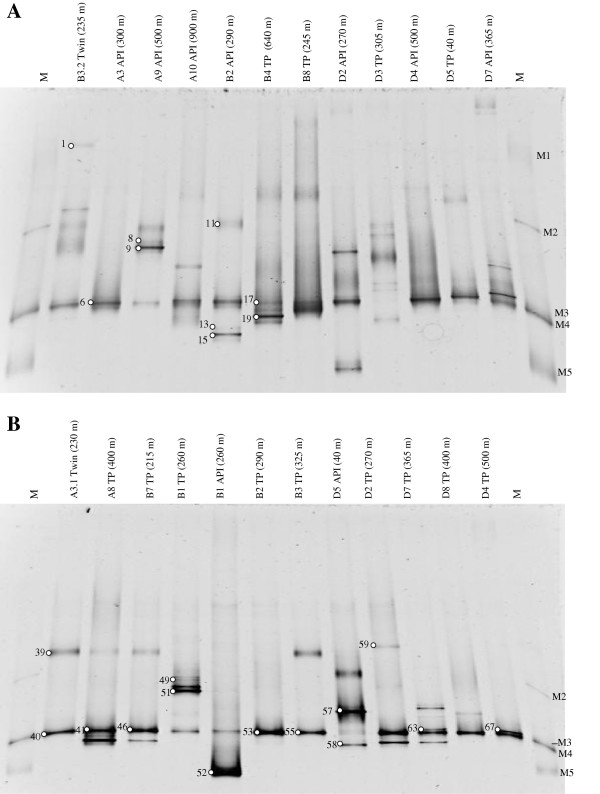
**16S RNA gene amplified from DNA isolated from cultures.** Separation of PCR products amplified with bacteria specific 16S rRNA primers in 6% polyacrylamide gels with 0–100% denaturing gradient. Panels **A** and **B**. 16S RNA gene amplified from DNA isolated from 24 cultures. The number above each lane indicates the culture name and the number in parenthesis represents depth of isolation in meters. Lanes M are the ladders: M1= *Aquimarina muelleri*, M2= *Escherichia coli*, M3= *Sulfurimonas denitrificans*, M4= *Vibrio alginolyticus* and M5= *Mycobacterium smegmatis*. The sequenced bands are identified by number.

Twenty-four cultures, in which black coloration with ferrous sulfide precipitation was observed, were selected for further investigation. Using TWIN pack medium, two cultures were obtained during 2005: one from station A and another from station B. Using TP medium, we obtained thirteen cultures during 2006: one from station A, six from station B, and six from station D. Using API medium, nine cultures were obtained during 2006: three from station A, two from station B and four from station D (Figure 1). All of the cultures had variable cell morphology, with curved rod-shaped bacteria with polar spores predominant.

### Molecular identification

**Figure 2:**
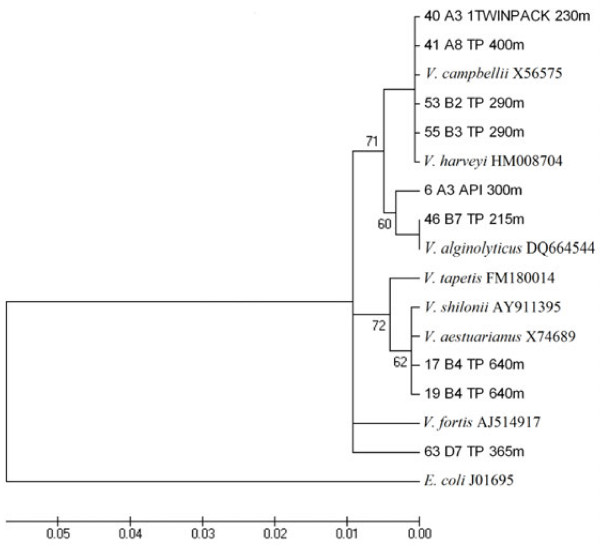
**Phylogenetic tree of partial 16S rRNA sequences of genus *Vibrio *isolated from the Cariaco Basin.** Tree was constructed using Neighbor-Joining algorithm. Bootstrap values are based on 1000 replicates and no values are given for groups with Bootstrap values less than 50%. The scale bar represents 0.01 (1%) nucleotide sequence difference.

All 16S RNA gene amplicons from the 24 cultures were separated in the DGGE gels (Figure 1). The patterns comprised several bands, suggesting that different bacterial types were present. Sixty-seven DGGE bands were excised and sequenced, but only 23 quality sequences were generated. These sequences were compared with existing sequences in the NCBI public database using the BLAST algorithm. Results of the BLAST analyses are summarized in Table 2. A prevalent band in the DGGE gels was observed for all samples, except D3 TP (305 m). Bands showing similar mobility gave highly similar or identical sequences with the molecular marker M4 (*Vibrio alginolyticus*) (Figure 1). Several of these bands (6, 40, 41, 46, 53, 55 and 63) were sequenced and shared 99-100% similarity with *Vibrio* species, forming a linkage with *V. campbelli*, *V. harveyi*, and *V. alginolyticus*. Band 63 was distant from the main *Vibrio* species cluster, appearing to be more closely related to *Vibrio fortis* (98%) (Figure 2). Additionally, two bands (17, 19) showed 96% similarity with the *Vibrio* cluster, forming a clade with *V. shilonii* and *V. aestuarianus* (Table 2 and Figure 2). These results indicate that *Vibrio* species are common in our enrichments from Cariaco Basin water column.

**Table 2:** Bacteria detected in the present study

**Station**	**Zone**	**Depth**	**Culture/ Sample**	**Band**	**closest Blast relative (GenBank accession number)**	**% identity**
A	Oxic	230 m	A3.1 TWIN	39	Uncultured bacterium clone 1NT1c10_D09 (GQ413739)	99
					Uncultured Bacteroidetes bacterium clone PG-16-1-2 (EU626578)	93
				40	*Vibrio parahaemolyticus* (EU155540)	99
	Interface	300 m	A3 API	6	*Vibrio* sp. (EU854873)	99
	Anoxic	400 m	A8 TP	41	*Vibrio parahaemolyticus* (GU064371)	99
		500 m	A9 API	8	Uncultured bacterium clone 1NT1c10_A05 (GQ413699).	99
					*Fusobacterium perfoetens* (M58684)	95
				9	Uncultured bacterium clone 1NT1c10_A05 (GQ413699)	99
					*Fusobacterium perfoetens* (M58684)	97
B	Oxic	215 m	B7 TP	46	*Vibrio* sp. (EU854855)	99
		235 m	B3 2 TWIN	1	Uncultured bacterium clone SGUS1101 (FJ202956)	99
					Uncultured Clostridia bacterium clone 4DP1-A6 (EU780347)	99
	Interface	260 m	B1 TP	49	Uncultured bacterium clone 1NT1c10_A05 (GQ413699)	99
					*Fusobacterium perfoetens* (M58684)	96
				51	Uncultured bacterium clone 1NT1c10_A05 (GQ413699)	99
					*Fusobacterium perfoetens* (M58684)	96
			B1 API	52	*Enterobacter cloacae* ATCC13047-T (AJ251469)	100
		290 m	B2 API	11	Uncultured bacterium clone RefT1c10 (GQ413678)	99
					*Marinifilum fragile* (FJ394546)	96
				13	*Desulfovibrio* sp. An30N (AB301719)	99
				15	*Desulfovibrio* sp. An30N (AB301719)	96
			B2 TP	53	*Vibrio harveyi* (HM008704)	99
	Anoxic	325 m	B3 TP	55	*Vibrio parahaemolyticus* (FJ547093)	100
		640 m	B4 TP	17	*Vibrio* sp. (GU223598)	99
				19	*Vibrio* sp. (EF587982)	98
D	Oxic	40 m	D5 API	57	*Spirochaeta* sp. Antartic (M87055)	95
				58	Uncultured bacterium clone L-D-2 (AB154510) *Desulfovibrio* sp.	98
	Interface	270 m	D2 TP	59	*Marinilabilia salmonicolor* strain AQBPPR1 (GU198996)	95
	Anoxic	365 m	D7 TP	63	*Vibrio* sp (FJ952814)	99
		500 m	D4 TP	67	*Shewanella* sp (GQ203107)	98

**Figure 3:**
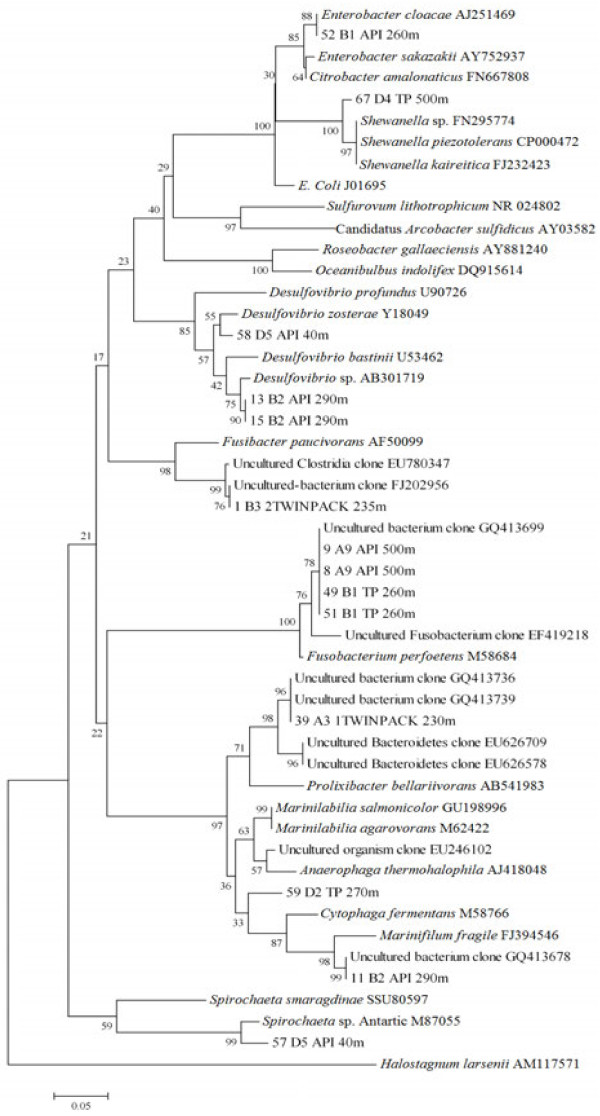
**Phylogenetic tree of partial 16S rRNA sequences obtained from the Cariaco Basin.** Tree was constructed using Neighbor-Joining algorithm. Bootstrap values are based on 1000 replicates. The scale bar represents 0.05 (5%) nucleotide sequence difference.

Three bands showing similar migration patterns had high sequence similarity (96–99%) with the 16S rRNA of bacteria belonging to the *Desulfovibrio* genus (Figure 3). Bands 13 and 15 clustered with a *Desulfovibrio* sp. isolated from a shallow submarine hydrothermal system in a tropical environment [13]. Band 58 had 99% similarity with an uncultured *Desulfovibrio* from sediment samples of a saline meromictic Lake in Japan [14] and clustered with *D. zosterae* at ≥ 96% similarity (Table 2 and Figure 3). The four other bands in the TP cultures from several depths (215–400 m), with similar patterns as *Desulfovibrio*, were observed (Figure 1) but not successfully sequenced. Over all, the results indicate that several *Desulfovibrio* species are present in the Cariaco water column at depths between 40 to 400 m (Table 2).

A representative of another genus located at the interface at station B and in the anoxic zone at station A was *Fusobacterium*, represented by four bands (Table 2). Bands 9, 49, and 51 showed 100% similarity and 99% with band 8, forming a group with an uncultured bacterium clone (GQ413699) and *Fusobacterium perfoetens* (Figure 3). Sequences belonging to *Enterobacter*, *Shewanella*, *Marinifilum*, *Mariniliabilia* and *Spirochaeta* were also identified in the Cariaco Basin water column enrichments (Table 2 and Figure 3). *Enterobacter cloacae*, a γ-proteobacteria was detected at 260 m in the interface zone of Station B. *Spirochaeta* sp., *Mariniliabilia* sp. and *Shewanella* sp. were found in the oxic (40 m), interface (270 m), and anoxic (500 m) zones, respectively, at Station D. Bands 63 (*Vibrio* sp.) and 67 (*Shewanella* sp.) had a similar migration pattern (Figure 1) and their sequences were 94% similar. Both, however, belong to γ-proteobacteria (Figure 3). Band 59 had 96% similarity with *Mariniliabilia*, an uncultured clone from a saltern evaporative lagoon in Puerto Rico, belonging to the Bacteroidales, while band 57 had 95% similarity with a *Spirochaeta* strain isolated in Antarctica (Figure 3).

Band 11 from the interface at station B (290 m) showed 96% similarity with *Marinifilum fragile* and 99% with an uncultured bacterium clone from coral reef samples in the Philippines [15]. The last of the bands that were sequenced (1 and 39) were from the oxic zone of Station A and matched with uncultivated clones most closely related to the Firmicutes and Bacteroidetes phyla (Table 2 and Figure 3). All eight genera detected in this study are strictly or facultative anaerobic bacteria with some relationship to sulfur cycling.

## Discussion

Isolation of the vast majority of bacteria in pure culture from the environment is hindered by lack of knowledge of specific culture conditions and by the potential synergism between organisms [16]. Recently, molecular approaches, such as rRNA analysis, have been used to determine bacterial species composition of microbial communities [16,17] and sequences of genes allow grouping and identification of the microorganisms. Genetic fingerprinting of microbial communities by DGGE provides banding patterns that reflect the genetic diversity of the community [16] or, as in this study, the diversity of a portion of the culturable community. DGGE of PCR-amplified gene fragments is one of the fingerprinting techniques used to separate fragments of identical length on the basis of primary sequence and base composition [16,17]. Different DGGE bands, indicating several different bacteria were detected and sequenced and the bands were identified as being derived from the genera *Vibrio*, *Desulfovibrio*, *Enterobacter*, *Shewanella*, *Fusobacterium*, *Marinifilum*, *Mariniliabilia* and *Spirochaeta*. Although 200bp sequence length can be considered too short for a phylogenetic analyses we founded that ours sequences correspond to genera, groups and classes like *Vibrio* sp., CFB group, gamma and delta proteobacteria that has been reported in the water column of the Cariaco basin previously [1,5,9,10,12].

The genus *Vibrio* encompasses a diverse group of heterotrophic marine bacteria and is widespread in the aquatic environment, occupying a variety of ecological niches. There are indications that vibrios play a role in nutrient cycling by taking up dissolved organic matter [18]. *Vibrio*-affiliated sequences were detected by DGGE gel analysis in the sample from both years (Figure 1) and 16S rRNA sequence similarity with of *V. campbellii*, *V. harveyi*, and *V. alginolyticus* (Figure 2). The *Vibrio* core cluster (including *Vibrio harveyi*, *V. alginolyticus* and *V. campbellii*) is often difficult to resolve solely by 16S rRNA gene heterogeneity, since species within the *V. harveyi* clade has a very high degree of both genetic and phenotypic similarity. These species have more than 99% sequence identity in the 16S rRNA gene [19,20].

*Vibrio harveyi* is found in a free-living state in aquatic environments and as part of the normal microbiota of marine animals. However, many variants of *V. harveyi* have been recognized as significant pathogens of aquacultured marine fish [21,22], crustaceans [23], lobsters [24], and corals [25]. Moreover, since 1993, *V. harveyi* has been recovered from diseased fish and penaeids in Venezuelan waters close to the Cariaco Basin (Paria Peninsula, Sucre State) [21].

Three bands 17, 19 and 63 had low 16S rRNA similarity (around 96-98%) with known vibrio species (Figure 2). Bands 17 and 19 were 96% similar to *Vibrio shilonii*. This species has been associated with healthy or necrotic corals in the Caribbean and Pacific reefs [18,26-30]. *V. shilonii* has recently reported in Cariaco Basin waters in the oxic layer using specific primers for Vibrio species in Station A [12] while we founded at 640 m depth (anoxic zone) in Station B. Band 63 is more closely related to *V*. *fortis* (98%). This species was detected directly in water samples between 200 m and 1300 m of the Station A in a previous report [12] were becomes a prominent *Vibrio* sp. in the redoxcline and anoxic zone. Furthermore, Raina *et al*. (2009) found *Vibrio* and *Shewanella* species to be able to degrade the sulfur compounds, e.g. DMSP, DMS and acrylic acid, associated with coral reef tissue and in the surrounding water, suggesting a role for these genera in the biogeochemical cycling of sulfur [31].

Expected was the finding of *Desulfovibrio* in the SRB culture (Figure 3). During the last two decades, an increasing number of novel sulfate-reducing bacteria have been isolated from a wide variety of environments, where strains of the genus *Desulfovibrio* are commonly found [32,33]. In this study, we detected *Desulfovibrio* species in the water column at 40 to 400 m depth, between the oxic and strictly anaerobic zones. Hastings and Emerson (1988) reported sulfate reduction in the presence of oxygen in and above the chemocline of the Cariaco basin and recent reports, using molecular techniques, showed sulfate reducing δ-proteobacteria cells were mainly associated with the oxic-anoxic interface zone and in the water column up to the aerobic zone (30 m) [1,5,9]. The *Desulfovibrio* phylotypes detected in this study were most similar to the uncultivated environmental clones of sulfate-reducing δ-proteobacteria and those mainly from tropical marine environments (Table 2).

Other bacteria identified among our cultures were *Enterobacter*, *Shewanella*, *Fusobacterium*, *Mariniliabilia* and *Spirochaeta* (Table 2 and Figure 3). *Spirochaeta* genus was report in sediments from Guaymas Basin [34]. *Enterobacter* sp., *Fusobacterium perfoetens* and *Spirochaeta* sp. are active in marine biocorrosion, formation of biofilms on carbon steel surfaces, and corrosion of oil field pipelines [35-37]. Our study showed four bands that were most similar to an uncultured bacterium clone related to *Fusobacterium* that had been isolated from coral reef samples [15]. *Shewanella* sp. has been associated with *Vibrio* sp., in the corrosion of carbon steel in saline media. These facultatively anaerobic bacteria can consume residual oxygen and thereby provide ecological niches for growth of SRB. Depending on environmental conditions, *Shewanella* sp. can produce hydrogen sulfate from elemental sulfur, reduce ferric iron and use cathodic hydrogen, competing with SRB for H_2_ as an energy source [38].

The genus *Marinilabilia* was created to include the marine, facultative anaerobic *Cytophaga* species, *Cytophaga salmonicolor* and *Cytophaga agarovorans*[39]. Taxonomic investigations have shown an overlap between the genera *Cytophaga* and *Flavobacterium* and these groups were then called the Cytophaga-Flavobacterium complex. Molecular investigations revealed an unexpected relationship between the *Cytophaga*-*Flavobacterium* group and the genus *Bacteroides* (CFB group) [39,40]. The CFB group had previously been reported to occur throughout the entire water column in the Cariaco Basin [1,9], in sediments from Guaymas Basin [34] and in anoxic cultures of rice paddy soil [41]. Our study showed that *Marinilabilia salmonicolor*, *Marinifilum fragile*, and an uncultured Bacteroidetes marine species of the CFB group were present, along with SRB, near the redox interface.

The lactate-sulfate media can enrich for SRB using lactate as an electron donor for the reduction of sulfate. However, other anaerobic or facultative microbes not reducing sulfate may also be found. Here we analyzed enrichments which showed the presence of a black FeS precipitate, indicating that some sulfate reduction must have occurred and found by DGGE other bacterial groups like ganma proteobacteria and CFB on those enrichments associated with *Desulfovibrio* species, showing the lack of specificity of lactate-sulfate media for SRB enrichment.

## Conclusions

Many studies have been conducted to identify the microorganisms present in the stratified environment of the Cariaco Basin and how they influence geochemical processes [1,3,5]. The high levels of sulfide present in the basin have been attributed to biological sulfate reduction [6]. However, very few studies have included enrichment for bacteria associated with sulfur cycling in this particular environment. In our studies, we showed an association of SRB-like *Desulfovibrio* with *Vibrio* species and other genera that have a previously defined relevant role in sulfur transformation and coupling of carbon and sulfur cycles in an environment where there are variable redox conditions and oxygen availability. This study provides new information about microbial species that were culturable under these conditions at several locations in the Cariaco Basin.

## Methods

### Sampling site and physico-chemical measurements

**Figure 4:**
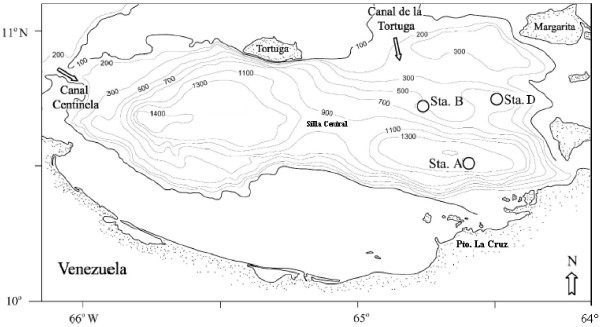
**Cariaco basin map.** Stations (Sta.) indicated by circles. Isobaths are in meters. Arrows indicate pathways of water intrusion from the Caribbean Sea. Sta. A: Station of oriental depression (10°30’N, 64°40’O); Sta. B: Station 700 m deep (10°40’N, 64°45’O); Sta. D: Station between the Araya and Cubagua sills (10°43’N, 64°32’O). This figure is adapted from Lin *et al*. (2008).

The Cariaco Basin is currently the focus of the CARIACO time series, a cooperative United States-Venezuelan research project (http://www.imars.usf.edu/CAR/), and is located on the Venezuelan continental shelf (Figure 4). The basin is isolated from the rest of the Caribbean Sea by a 150 m deep sill connecting Isla Margarita to Cabo Codera on the Venezuelan mainland [11,42].

Five hundred milliliters of seawater were collected at different depths (40–230, 260–300, and 325–640 m for oxic, interfase and anoxic water column zones) on May 25–27, 2005, and May 19–20, 2006 (CAR-112 and 122, respectively), at three locations (Figure 4), including the CARIACO time-series station (station A: 10°30′N 64°40'W), a station southeast of La Tortuga Channel in water about 600 m deep (station B: 10°40′ N 64°45'W), and a station between the Cubagua and Araya sills (station D: 10°43'N 64°32'W).

Water column sampling was conducted aboard the R/V Hermano Gines, operated by Estación de Investigaciones Marinas (EDIMAR), Fundación la Salle de Ciencias Naturales, Margarita Island, Venezuela and samples were collected with a SeaBird rosette, accommodating 12 TFE-lined, 8-L Niskin bottles. Profiles of temperature, salinity, and O_2_ were obtained with a Seabird conductivity-temperature-depth (CTD) system with attached SBE 43 oxygen probe. The Niskin bottles were slightly pressurized with N_2_ during sampling to minimize contact with O_2_. Based on redox conditions and oxygen content for the sampling cruises, oxic layer was considered between (~ 0–240 m); redox transition interface (~ 245–320 m); and anoxic layer (~ 325 to 900 m).

### Culture conditions

Cultivation of SRB in sealed serum bottles containing filter sterilized seawater amended with three different culture media: Twin Pack (Twin) medium (per liter of distilled water: K_2_HPO_4_, 2.00 g; MgSO_4_•7H_2_O, 0.10 g; CaCl_2_•2H_2_O, 0.10 g; ammonium sulphate, 0.10 g; FeCl_3_, 0.02 g; sodium thiosulfate, 10.0 g; pH adjusted at 7,8 ±0,2) supplemented with 0.2% lactate; Triple Pack (TP) medium (HIMEDIA, India) supplemented with 0.2% lactate as a carbon source and a solution of ferrous ammonium sulfate (0.0392 mg/L) and sodium ascorbate (0.01 mg/L) as reducing agent; and Modified American Petroleum Institute (API) medium (HIMEDIA, India), supplemented with ascorbic acid (0.1 g/L) as a reducing agent [43] and using 0.5% acetate as a carbon source. Each culture medium was inoculated with 10 mL of 20 water samples (6 samples for 2005; 14 samples for 2006) by duplicate and incubated at room temperature for 30 days under a gas mixture consisting of 20% CO_2_: 80% N_2_. 120 cultures were performed in total.

### DNA extraction and PCR

DNA was extracted from the Twin, TP, and API cultures using the Microbial DNA Isolation kit (Mo Bio Laboratories, CA, USA), according to manufacturer’s recommendation. Bacterial DNA was amplified using a primer with GC clamp (341F-GC: 5'-CGC CCG CCG CGC GCG GCG GGC GGG GCG GGG GCA CGG GGG GCC TAC GGG AGG CAG CAG-3') and 907R (5'-CCG TCA ATT CGT TTG AGT TT-3') [16,44]. The reaction mixture contained 3 μL of DNA (approximately ~50-100 ng) and 0.5 μM of each primer, 35 μL of GoTaq Green Master Mix reactions (Promega, Madison, WI, USA) and water added to a final volume of 70 μL. PCR amplification was performed in a thermal cycler (PxE Thermal Cycler, Thermo Hybaid, IL, USA), as follows: 95°C for 5 min; 20 cycles at 94°C for 30 s; 65°C for 1 min; 72°C for 3 min; 15 cycles at 94°C for 30 s; 55°C for 1 min; 72°C for 3 min; and 72°C for 7 min. The negative PCR control had no template in the reaction. The positive control for PCR was prepared by adding 1 μL of *Alcaligenes faecalis* DNA (100 ng). The PCR products were visualized by running the reaction mixture in a TBE agarose gel (1.0%), staining with ethidium bromide (0.2 μg/ml), and observing under UV light.

### Denaturing gradient gel electrophoresis (DGGE)

DGGE analysis of the bacterial amplicons (70 μL - entire volume of a PCR reaction) was performed in 6% polyacrylamide (37.5: 1 acrylamide/bis-acrylamide) gels containing a 0–100% urea plus formamide gradient (100% denaturing solution containing 7 M urea and 40% (v/v) formamide).

Electrophoresis was performed in 0.5 X TAE (TRIS acetate 20 mM [pH 7.41], sodium acetate 10mM, and sodium EDTA 0.5 mM) at 60 volts and 60°C for 14 h using a DGGE 1001–110 System (C.B.S. Scientific Company, Inc). Gels were stained with ethidium bromide (0.2 μg/mL) for 20 min and visualized using a FOTO/Analyst Investigator/FX Systems (Fotodyne Incorporated, Hartland, WI, USA) [12].

### 16S RNA gene sequence analysis

Separated DNA fragments were excised from the DGGE gels, placed in a freezer at −80°C for 2 h, and blended in Mini-Beadbeater 8 (BioCold Scientific, Fenton, MO, USA), for 3 min with 0.2 g sterile zirconia/silica beads (BioSpec Products, Bartlesville, OK) in 500 μL sterile HPLC water (Fisher HealthCare). Samples were stored at 4°C overnight, after which 3 μl aliquots were used as template for PCR amplification of 16S RNA gene, employing primers 341F (same as 341F-GC but without GC clamp) and 907R and the same PCR conditions as described above, with a final PCR volume of 50 μL.

Re-amplified PCR products were purified using a Wizard SV gel and PCR clean-up system kit (Promega, Madison, WI, USA). Sequencing of one DNA strand was performed using the BigDye^TM^ Terminator v3.1 sequencing kit, following manufacturer’s instructions (Applied Biosystems, Foster City, CA). Sequencing reactions were analyzed in a 3100 ABI DNA sequencer and sequence quality was determined using Chromas Lite software (http://www.technelysium.com.au/chromas_lite.html) [12].

The closest known relatives of the partial 16S RNA gene sequences were identified using BLASTN 2.2.1 (http://www.ncbi.nlm.nih.gov/blast/) [45].

### Phylogenetic analysis

Partial 16S rRNA gene sequences initially were compared with sequences in the GenBank database using BLASTN [45] to determine their approximate phylogenetic affiliation. Environmental sequences, together with closest GenBank matches, were aligned in http://greengenes.lbl.gov using the NAST Alignment utility [46]. Sequences obtained from 23 DGGE bands were aligned using NAST Alignment [46], and a phylogenetic tree was constructed using 200 bp long aligned sequences and the neighbor-joining algorithm (Jukes-Cantor Model) in Molecular Evolutionary Genetics Analysis 2.1 software (MEGA, version 4) [47]. Bootstrapping was used to estimate reliability of the phylogenetic reconstructions (1000 replicates). Representative sequences were submitted to GenBank database and are designated by accession numbers HM466893-HM466915.

## Competing interests

The authors declare that they have no competing interests.

## Authors’ contributions

LBH participated in the design of the study, carried out the sampling collection and molecular genetic studies, participated in the phylogenetic analyses and the sequence alignment, and co-wrote the manuscript. MAG participated in the phylogenetic analyses of the samples and the sequence alignment, and co-wrote the manuscript. AC participated in the design of the study and coordination of the molecular genetic studies. RV and JJN compiled the physic-chemical data related to the Cariaco Basin and B/O Hermano Gines logistic and sampling collection. RC drafts the manuscript and improves the discussion of the results. PS participated in the design of the study, carried out the sampling collection and the phylogenetic analyses, and co-wrote the manuscript. All authors read and approved the final manuscript.
